# ﻿*Gastrodiabawanglingensis* (Orchidaceae, Epidendroideae), a new species from Hainan Island, China

**DOI:** 10.3897/phytokeys.220.95137

**Published:** 2023-02-23

**Authors:** Zhi-Heng Chen, Zhong-Yang Zhang, Xi-Qiang Song, Zhe Zhang

**Affiliations:** 1 Key Laboratory of Genetics and Germplasm Innovation of Tropical Special Forest Trees and Ornamental Plants, Ministry of Education, Hainan University, Haikou 570228, China; 2 College of Forestry, Hainan University, Haikou 570228, China; 3 Key Laboratory of Biology of Tropical Flower Resources of Hainan Province, Haikou 570228, China

**Keywords:** Gastrodieae, Hainan Tropical Rainforest National Park, holomycotrophic orchids, taxonomy, tropical rainforest

## Abstract

*Gastrodiabawanglingensis*, a new species of Orchidaceae from Hainan Island, China, is described and illustrated. It is morphologically similar to *G.theana*, *G.albidoides* and *G.albida* with dwarf habits, scarcely opening flowers, elongated fruit stems, curved and fleshy perianth tubes and similar columns and lips, but can be easily distinguished from them by having a pair of lateral wings bent outwards at the apex of the column and lateral wings with acuminate tips lower than the anther. According to the IUCN Red List Categories and Criteria, the new species is assessed as Endangered (EN). The plastome of *G.bawanglingensis* is greatly reduced and reconfigured with approximately 30876 bp in size and 25.36% in GC content. Morphological characteristics and molecular phylogenetic results based on chloroplast gene sequences support the recognition of *G.bawanglingensis* as a new species within *Gastrodia*.

## ﻿Introduction

**Figure 1.**
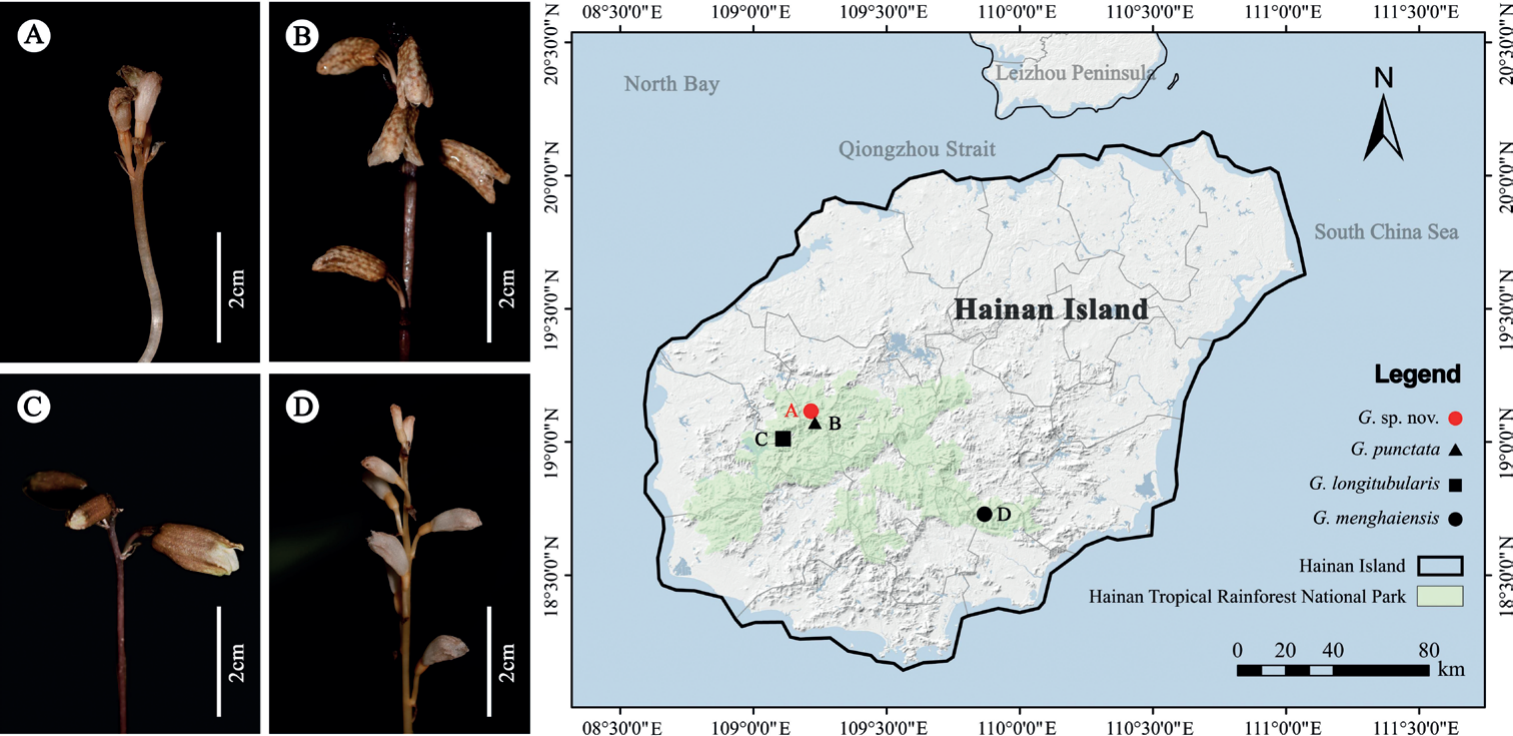
Pictures and distribution of *Gastrodia* species in Hainan Island based on our field investigation in the past three years. G., *Gastrodia***A***G.* sp. nov. **B***G.punctata***C***G.longitubularis***D***G.menghaiensis*.

*Gastrodia*Brown (1810: 330) (Epidendroideae, Gastrodieae) comprises approximately 100 species and is widespread from northeast India through the eastern Himalayas and southern China to Japan and eastern Siberia, southwards to Malaysia and Australia, eastwards to the Pacific Islands as far as Samoa and westwards to Madagascar, Mascarene Islands and tropical Africa (Pridgeon et al. 2005; Chen et al. 2009; Cribb et al. 2010; Chase et al. 2015; Jin and Kyaw 2017; Suetsugu 2019, 2021; Bandara et al. 2020; Liu et al. 2021). There are 33 known species (16 endemic) of *Gastrodia* in China, mainly distributed in southern China, including Tibet, Fujian, Hainan, Yunnan, Sichuan and Taiwan (Liu et al. 2021; Zhou et al. 2021). In Hainan Island, three species, namely *Gastrodialongitubularis* Q.W.Meng, X.Q.Song & Y.B.Luo (Meng et al. 2007), *G.punctata* Aver. (Lu et al. 2017) and *G.menghaiensis* Z.H.Tsi & S.C.Chen (Huang et al. 2021), have been reported from the tropical rainforest (Fig. 1).

During our field investigation in April 2021, *Gastrodia* specimens with significantly different floral morphology from all the known species in China were collected in the forests of Bawangling, Hainan Tropical Rainforest National Park. Further studies, based on examination of specimens and literature of *Gastrodia* (Averyanov 2005; Hsu and Kuo 2011; Tan et al. 2012) and comparison with type specimens, showed that those specimens represent a new species that is morphologically distinct from previously-known taxa of the genus *Gastrodia* and is described below.

## ﻿Materials and methods

### ﻿DNA extraction and sequencing

The next generation sequencing technology (high-throughput sequencing) was applied to extract the total genomic DNA of plant materials and chloroplast splicing software GetOrganelle was used to assemble the plant genome (Jin et al. 2020). Moreover, online annotation software Geseq (https://chlorobox.mpimp-golm.mpg.de/geseq.html) (Tillich et al. 2017) and CpGAVAS (http://www.herbalgeno-mics.org/cpgavas) (Liu et al. 2012) were used to determine the chloroplast genome start position and IR region and annotate the genes on the chloroplast genome. Finally, we used manual proofreading to verify the correctness of the annotations, according to the reference of NC_024662.1.

### ﻿Phylogenetic analysis

**Figure 2.**
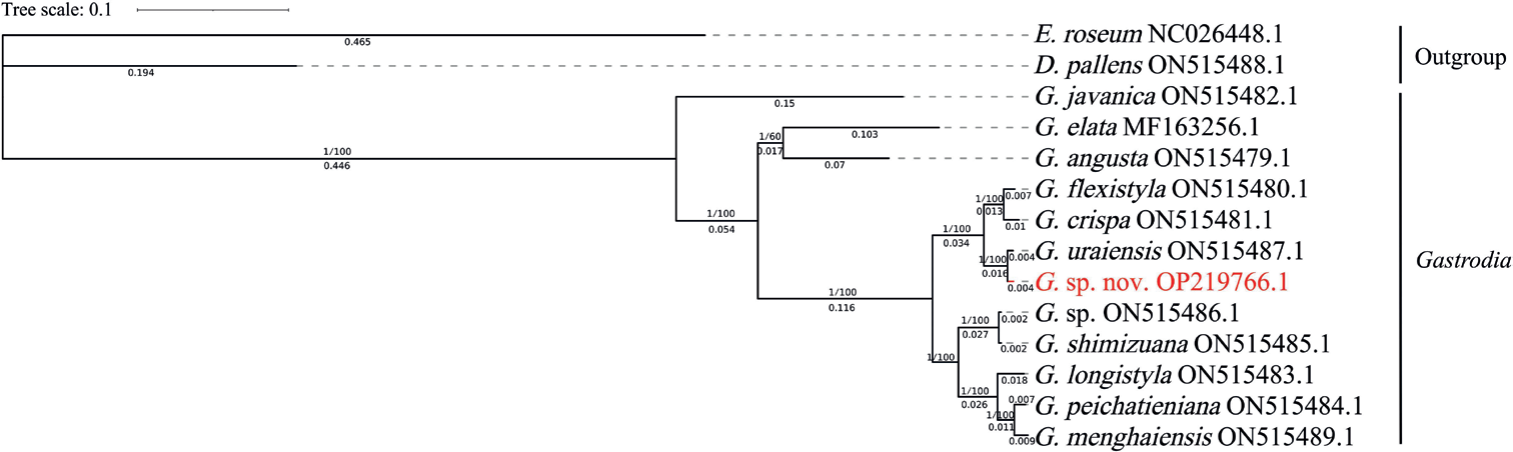
Phylogenetic tree reconstruction of *Gastrodia* using the maximum likelihood (ML) method based on chloroplast gene sequences of *Gastrodia* sp. nov. and 11 other species. Only the ML tree is shown, because its topology is nearly identical to that of the obtained BI tree. Numbers associated with the branches are BI posterior probabilities (PP) and ML bootstrap value (BS). The species name is followed by the accession number of the GenBank accession. D, *Didymoplexis*; E, *Epipogium*; G, *Gastrodia*.

To estimate the phylogenetic position of the *Gastrodia* sp. nov. within *Gastrodia*, phylogenies were reconstructed by Maximum likelihood (ML) and Bayesian Inference (BI) analyses using the coding sequences (CDSs). All plastomes were downloaded from the NCBI database except *Gastrodia* sp. nov. (Wen et al. 2022). In the phylogenetic tree, *Epipogiumroseum* (D.Don) Lindl. and *Didymoplexispallens* Griff. were selected as outgroup; *Epipogium* belongs to Nervilieae, a sister tribe to Gastrodieae while *Didymoplexis* is sister to *Gastrodia* (Wen et al. 2022). The sequences of the species and related ones were aligned in MAFFT version 7 (https://mafft.cbrc.jp/alignment/server/) using MAFFT (Katoh and Standley 2013) by default setting. Phylogenetic construction was conducted by Maximum Likelihood with MEGA11 software (Tamura et al. 2021), selecting the best-fit model of GTR+G with 1000 bootstraps (Nei and Kumar 2000), and Bayesian Inference (BI) tree in MrBayes 3.2.7 using the GTR+G model (Ronquist et al. 2012), runs for 20 million generations. Phylogenetic trees were sampled every one thousand generations, the first 25% of trees generated were discarded as burn-in and the remaining trees were used to construct majority-rule consensus tree. Finally, the tree file was visualised and annotated on iTOL (https://itol.embl.de/) (Ivica and Peer 2021). All the sequences’ accession numbers were listed in Fig. 2.

### ﻿Morphological description

Morphological observations of *Gastrodia* sp. nov. were based on living plants (four individuals) and dried herbarium specimens all belonging to the type specimen, which is kept in the HUFB (Teaching Herbarium of the College of Forestry, Hainan University). All length and width of structures were measured by vernier calipers. Morphological characters of the new species were based on dried herbarium specimens. Furthermore, we examined the type specimens of *Gastrodiaalbidoides* Y.H.Tan & T.C.Hsu, which is the most morphologically similar species to *Gastrodia* sp. nov. and housed in HFTC. High resolution photographs of living plants were provided by Zhong-Yang Zhang and Zhi-Heng Chen.

## ﻿Results

### ﻿Plastome of *Gastrodia* sp. nov.

**Figure 3.**
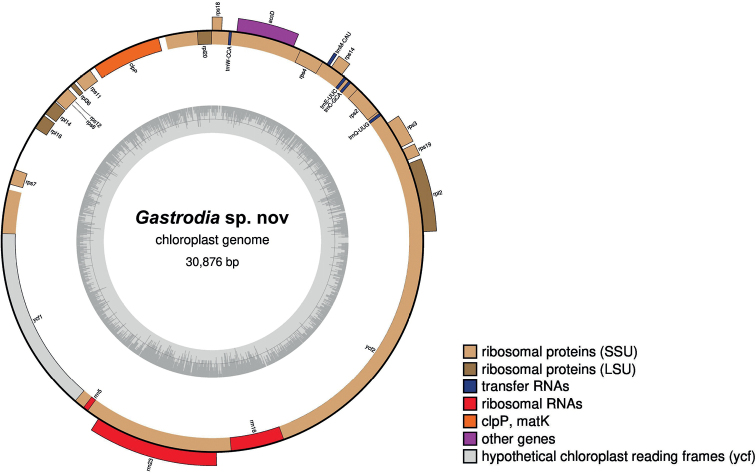
Plastome of *Gastrodia* sp. nov.

The plastome of the novelty is 30876 bp in length with its GC content approximately 25.36% (GenBank accession number: OP219766) (Fig. 3), which is similar to the 11 other species of *Gastrodia* (29,696–36,812 bp, Table 1). The plastome contains 19 protein-coding genes, five transfer RNA and three ribosomal RNA genes. Several genes and typical plastome regions appear to have been either lost or pseudogenised in *G.* sp. nov. The *G.* sp. nov. plastome does not contain housekeeping genes and lacks an IR region. This indicates that plastomes of *Gastrodia* are in the last stages of plastome degradation (see Barrett and Davis 2012; Liu et al. 2021; Jiang et al. 2022; Wen et al. 2022).

**Table 1.** Information on the chloroplast genomes of *Gastrodia* sp. nov. and other 11 species of *Gastrodia*.

Species	Length of chloroplast genome (bp)	GC content (%)	Number of genes		
			Protein coding genes	*t*RNA genes	*r*RNA genes
*Gastrodiaangusta*	36.812	25.4	19	5	4
*Gastrodiacrispa*	30.582	25.7	19	5	4
*Gastrodiaelata*	35.304	26.8	20	5	3
*Gastrodiaflexistyla*	30.797	25.4	19	5	4
*Gastrodiajavanica*	31.896	24.8	18	4	4
*Gastrodialongistyla*	30.464	24.8	18	5	3
*Gastrodiamenghaiensis*	30.118	24.9	19	4	3
*Gastrodiapeichatieniana*	29.696	25.9	18	5	4
*Gastrodiashimizuana*	30.019	25.5	18	5	4
*Gastrodia* sp.	29.944	25.8	18	5	4
*Gastrodia* sp. nov.	30.876	25.4	19	5	3
*Gastrodiauraiensis*	30.746	24.9	19	5	4

### ﻿Phylogenetic analysis

Our ML and BI phylogenetic trees constructed from the chloroplast gene sequences showed that the novelty belongs to the genus *Gastrodia*, and is related to *G.uraiensis*, *G.flexistyla* and *G.crispa*.

### ﻿Taxonomic treatment

#### ﻿ Gastrodia bawanglingensis

Taxon classificationPlantaeAsparagalesOrchidaceae

Z.H.Chen, Z.Y.Zhang & X.Q.Song sp. nov.

FE223882-DB1F-505A-B657-A97A4E716201

urn:lsid:ipni.org:names:77314677-1

Fig. 4

##### Type.

China. Hainan Province: Bawangling, Hainan Tropical Rainforest National Park, in tropical rainforest, 850–950 m elevation, 25 April 2022, Z.Y. Zhang 006 (Holotype, HUFB!).

##### Diagnosis.

*Gastrodiabawanglingensis* is similar to *G.albidoides* with dwarf habits, scarcely opening flowers, elongated fruit stems, curved and fleshy perianth tubes and similar columns and lips, but can be easily distinguished from the latter by having lateral sepals adnate to 4/5 of total length (vs. lateral sepals adnate to 1/2 of total length), lip with four ridges (vs. lip with two ridges), the absence of a column foot (vs. the presence of a column foot) and a pair of lateral wings bent outwards (vs. lateral wings upright) at the column apex (Table 2).

**Figure 4.**
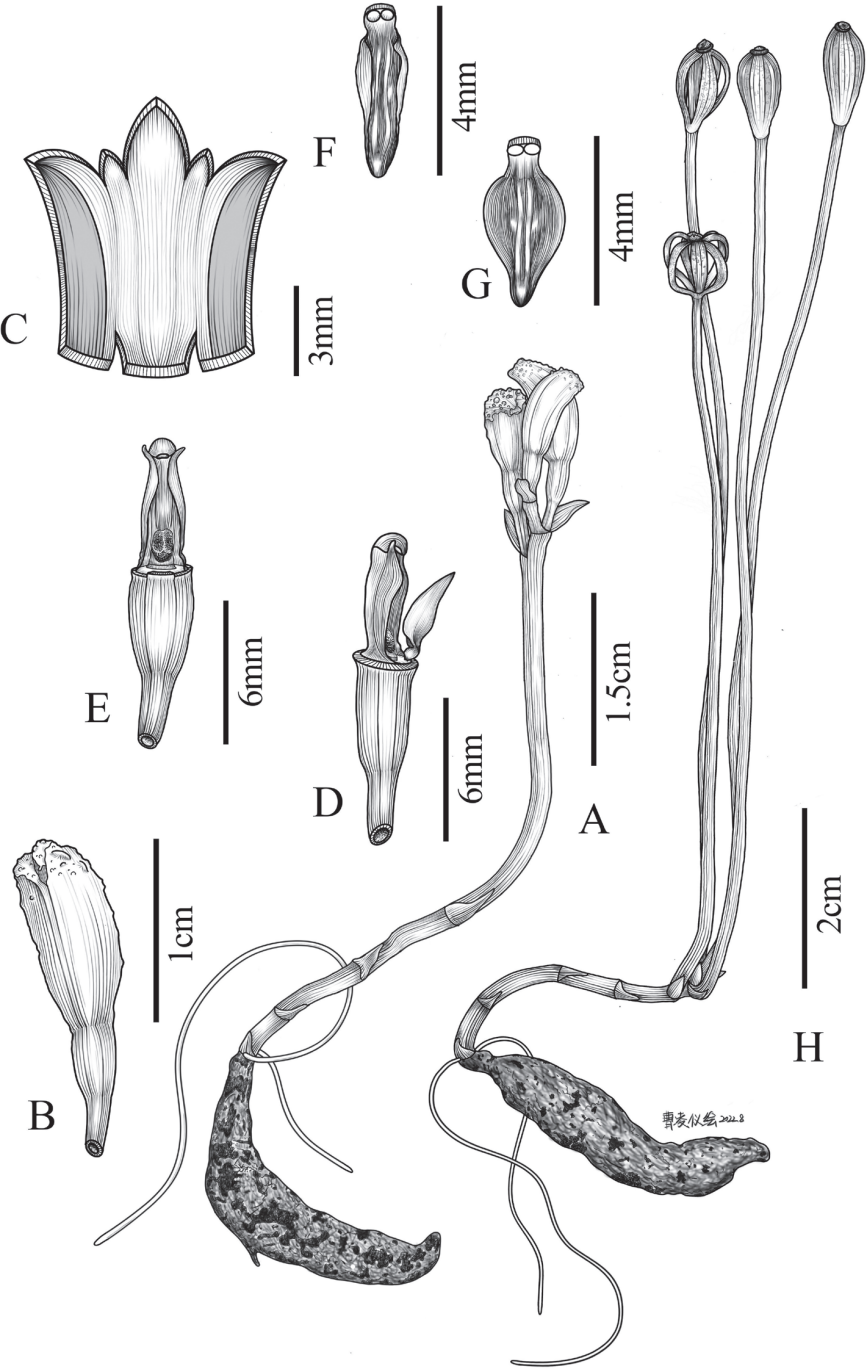
*Gastrodiabawanglingensis* Z.H.Chen, Z.Y.Zhang & X.Q.Song, sp. nov. **A** plant **B** flowers **C** flattened perianth tube **D** lip, column and ovary **E** column **F, G** lip **H** fruiting specimen. Illustration by Ling-Yi Cao, based on the holotype of Z.Y. Zhang 006 (HUFB).

**Table 2.** Differences between *Gastrodiabawanglingensis*, *G.albidoides*, *G.theana* and *G.albida*.

Character	*G.bawanglingensis*	*G.albidoides*	*G.theana*	*G.albida*
Perianth tube	slightly verrucous in the middle and upper part, distinct verrucose apically	slightly verrucose towards apex, otherwise smooth	distinctly striate and verrucose throughout	distinctly verrucose throughout
Lateral sepals	adnate, to 4/5 of their length	adnate, to 1/2 their length	adnate, 1/3–1/4 their length	adnate, 1/5–1/6 their length
Petals	brownish, fleshy, petals whitish on both sides, triangular-ovate, ca. 1.5 × 1.0 mm	whitish, thin in texture, triangular-ovate, 0.8–1.0 × 0.6–0.8 mm	salmon-pink, thin in texture, narrowly triangular, 0.4–0.8 × 0.2–0.3 mm	whitish outside, orange inside, fleshy, oblong-ovate, ca. 1.5 × 1.0 mm
Lip	red at the base, light green at the middle, reddish-brown apically and marginally, epichile rhombic-ovate, 5–7 nerved, disc thickened with four ridges, a pair of low ridges outside the two main ridges. truncate at base, hypochile with two whitish, globose, subsessile, nectarless calli, ca. 0.5 mm in diameter	pale green, epichile rhombic-ovate, 6–7-nerved, disc thickened with two ridges, rounded at base, hypochile with two whitish, globose, subsessile, nectarless calli, ca.1 mm in diameter	green, epichile ovate, 5-nerved, disc slightly elevate longitudinally at middle, with four ridges four ridges, arranged one behind the other. cordate at base, hypochile with two whitish, globose, subsessile, nectarless calli, ca.0.8 mm in diameter	white, epichile triangular, disc thickened with two ridges, truncate at base; hypochile with two whitish, globose, subsessile, nectarless calli, ca.1 mm in diameter
Column	apex with a pair of lateral wings bent outwards; lateral wings with acuminate tips lower than anther	apex with a pair of lateral wings; lateral wings with acuminate tips superior to anther	apex with a pair of lateral wings bent inwards; lateral wings with acuminate tips superior to anther	with a pair of lateral wings distally; edges of lateral wings parallel to column
Column foot	Absent	1.5–1.8 mm	1.5–1.8 mm	column foot very short
Rostellum	0.2 × 1.0 mm	0.2 × 1.5 mm	0.2 × 1.5 mm	Absent

##### Description.

Terrestrial, leafless, achlorophyllous herbs. Roots few, slender, 1–7 cm long, ca. 0.5–0.7 mm in diameter. Rhizome fleshy, tuberous, fusiform, 3–4 cm long, 5–7 mm in diameter, dark brown, covered with numerous scales. Scales verticillate, lanceolate, dark brown,1–2 mm long. Inflorescence erect, terminal, 2.0–6.5 cm long, ca. 2.2 mm in diameter, white to orange-brown, peduncle 3–4 noded, ovate to broadly ovate, sheath membranous, 3–5 × 2–3 mm; rachis often less than 5 mm long. Bracts membranous, ovate to ovate-oblong, apex pointed, pale yellowish-brown, 4–6 mm long, 1.5–3 mm wide. Ovary 3–6 mm long, 2–3 mm in diameter. Flowers (1–) 2–4 (–6), erect, bell-shaped, slightly curved, not opening widely, 8–10 mm long, 4–5 mm in diameter. Flowers whitish on both surfaces, apex brownish, lip red at the base, light green at the middle, reddish-brown apically and marginally; column white. Sepals and petals united, forming a 5–lobed perianth tube, 8–10 mm long, slightly verrucous in the middle and upper part, distinctly verrucose apically. Sepals fleshy, thickened, similar. Lateral sepals fused to 4/5 of their length, whitish on both surfaces, apex is brownish; free lobe of dorsal sepal triangle, ca. 2.5 × 2.0 mm; free lobes of lateral sepals ovate, ca. 2.0 × 2.0 mm. Petals connate with sepals, free portions brownish, whitish on both sides, triangular-ovate, ca. 1.5 mm long, 1 mm wide, connate portions distinctly thickened and the inside is obviously reddish-brown, forming a pair of ridge-like structures inside the perianth tube and the other side of the ridge-like structures is flesh-coloured. Lip rhombic-ovate, base adnate to perianth tube, 3.5–4.5 × 2.0–2.2 mm; hypochile with two whitish, globose, subsessile, nectarless calli, ca. 0.5 mm in diameter; epichile 5–7 nerved, truncate at base, entire, disc thickened with four ridges, a pair of low ridges outside the two main ridges; the two main ridges fused into one before reaching the tip, main ridges much raised and tinged orange near apex. Column 4.2–4.5 × 1.6–1.8 mm, apex with a pair of lateral wings bent outwards; lateral wings with acuminate tips lower than anther; column foot absent; rostellum 0.2 × 1 mm; stigma located near base. Anther hemispherical, 0.6–0.7 mm in diameter; pollinia 2. Capsule ellipsoid, 1.2–1.8 cm long, 0.5–0.8 cm in diameter; pedicel elongating to 10–25 cm in fruit. Seeds fusiform, 1.6–2.2 mm long.

##### Etymology.

The new species is named after Bawangling, the mid-west State of Hainan Island where it was discovered in a vast area of primitive montane rainforest.

##### Vernacular name.

霸王岭天麻 (Chinese pinyin: bà wáng lǐng tiān má).

##### Distribution and habitat.

*Gastrodiabawanglingensis* is a terrestrial mycoheterotrophic species that grows in montane rainforests which are dominated by *Dysoxylumgotadhora* (Buch.-Ham.) Mabb., *Livistonasaribus* (Lour.) Merr. and A.Chev., *Hanceahookeriana* Seem. and *Engelhardiaroxburghiana* Lindl. at elevations from 850 m to 950 m and associated with other orchids, such as *Anoectochilushainanensis* H.Z.Tian, F.W.Xing & L.Li, *A.roxburghii* (Wall.) Lindl., *Oxystophyllumchangjiangense* (S.J.Cheng & C.Z.Tang) M.A.Clem., *Dendrobiumhainanense* Rolfe, *Cymbidiumkanran* Makino and *Microperapoilanei* (Guillaumin) Garay. So far, only the type subpopulation has been found in the tropical rainforest of Bawangling, in Hainan.

##### Conservation status.

Endangered [EN D1]. *Gastrodiabawanglingensis* was discovered in the mountain rainforest of Bawangling in Hainan Tropical Rainforest National Park. Until now, only the type subpopulation, consisting of ca. 100 individuals, has been discovered in Bawangling. Since its number of mature individuals is fewer than 250, we assess it as Endangered (EN) using criterion D1 (IUCN Standards and Petitions Subcommittee 2022).

##### Phenology.

*Gastrodiabawanglingensis* was observed flowering and fruiting in April and May.

##### Pollination implication.

Flowers of *Gastrodiabawanglingensis* barely open and pollen massulae were observed on the stigma when flowers were dissected. Through field observation, it was found that the fruiting rate is very high. We bagged buds on 3 plants with 10 flowers in total prior to the anthesis, and found that each of them has evolved into fruit after 15 days. These observations indicate that the new species probably self-pollinates. *Gastrodia* is probably the only genus that contains species with completely cleistogamous flowers as confirmed by intensive monitoring. Self-pollination might be an adaptation to ensure reproduction, compensating for the defi- ciency of pollinators in the habitat (Suetsugu 2022; Suetsugu et al. 2022). Currently, complete cleistogamy has been reported in five *Gastrodia* species: *G.clausa*, *G.takeshimensis*, *G.flexistyloides*, *G.kuroshimensis* and *G.amamiana* (Hsu et al. 2012; Suetsugu 2013, 2014, 2016, 2019), *G.bawanglingensis* is likely to be the sixth species reported. Similar to other five species, *G.bawanglingensis* is also distributed on the island, further confirming island colonization may be one of the factors of evolution of complete cleistogamy. And compared with the mainland, there are more frequent geological and climate changes on the island, which may cause the rapid change of its living environment and lead to the loss of pollinators in its distribution area. Unreliable pollinator services and the cost of maintaining open flowers probably drove the completely cleistogamous *Gastrodia* species to abandon insect-mediated pollination (Suetsugu 2014, 2016). However, complete cleistogamy has arguably driven speciation (Kishikawa et al. 2019; Ogaki et al. 2019). We also found several other unpublished species that are different but very similar to *G.bawanglingensis* in our field survey in Hainan Island, which also confirms the above point of view. It is also notable that although lack of rostellum often facilitates selfing in the genus (Suetsugu 2022; Suetsugu et al. 2022), the new species has somewhat well-developed rostellum. Further observations are needed on how the species accomplishes autogamy.

## ﻿Discussion

*Gastrodiabawanglingensis* is most similar to *G.albidoides* (Tan et al. 2012) from Yunnan, *G.theana* (Averyanov 2005) from Vietnam and *G.albida* (Hsu and Kuo 2011) from Taiwan. They share dwarf habits, scarcely opening flowers, fleshy curved perianth tubes with verruca and similar columns and lips. After comparison of available literature and specimens, we conclude that *G.bawanglingensis* could be clearly differentiated from *G.albidoides*, *G.theana* and *G.albida* by several floral characters (Table 2).

### ﻿Key to the species of *Gastrodia* found in Hainan Island, China

**Table:** 

1	Sepals adnate to 4/5 of their length; lip light green at the middle, reddish-brown apically and marginally; lip disc with two ridges ranging from base to apex	***G.bawanglingensis***
–	Sepals adnate up to 1/2 of their length; lip green or white at the middle, uniform coloured or orange-red towards apex; lip disc without distinct ridges, but with lamellae or keel	**2**
2	Flowers white, sub-erect; petals margin wrinkled; column foot very short, pedicel elongated in fruit	***G.menghaiensis***
–	Flowers grey-brownish, horizontal or slightly bending; petals margin entire, column foot distinct; pedicel not elongated in fruit	**3**
3	Tepal tube without white spots; column cylindrical and thick; lip disc with a pair of longitudinal lamellae near apex	***G.longitubularis***
–	Tepal tube with white spots; column flat and thin; lip disc with four keels	***G.punctata***

## Supplementary Material

XML Treatment for Gastrodia bawanglingensis
